# The early-stage comprehensive costs of routine PrEP implementation and scale-up in Zambia

**DOI:** 10.1371/journal.pgph.0001246

**Published:** 2022-11-02

**Authors:** Cheryl Hendrickson, Lawrence C. Long, Craig van Rensburg, Cassidy W. Claassen, Mwansa Njelesani, Crispin Moyo, Lloyd Mulenga, Heidi O’Bra, Colin A. Russell, Brooke E. Nichols

**Affiliations:** 1 Health Economics and Epidemiology Research Office, Johannesburg, South Africa; 2 Department of Internal Medicine, School of Clinical Medicine, Faculty of Health Sciences, University of the Witwatersrand, Johannesburg, South Africa; 3 Department of Medical Microbiology, Amsterdam University Medical Center, Amsterdam, The Netherlands; 4 Department of Global Health, School of Public Health, Boston University, Boston, Massachusetts, United States of America; 5 Center for International Health, Education, and Biosecurity (CIHEB), Institute of Human Virology, University of Maryland School of Medicine, Lusaka, Zambia; 6 USAID DISCOVER-Health, John Snow, Inc. Lusaka, Zambia; 7 EQUIP, Lusaka, Zambia; 8 Ministry of Health, Lusaka, Zambia; 9 United States Agency for International Development, Lusaka, Zambia; Bangladesh Institute of Development Studies, BANGLADESH

## Abstract

Pre-exposure prophylaxis (PrEP) is an effective HIV prevention option, but cost-effectiveness is sensitive to implementation and program costs. Studies indicate that, in addition to direct delivery cost, PrEP provision requires substantial demand creation and client support to encourage PrEP initiation and persistence. We estimated the cost of providing PrEP in Zambia through different PrEP delivery models. Taking a guidelines-based approach for visits, labs and drugs, we estimated the annual cost of providing PrEP per client for five delivery models: one focused on key populations (men-who-have-sex-with-men (MSM) and female sex workers (FSW), one on adolescent girls and young women (AGYW), and three integrated programs (operated within HIV counselling and testing services at primary healthcare centres). Program start-up and support costs were based on program expenditure data and number of PrEP sites and clients in 2018. PrEP clinic visit costs were based on micro-costing at two PrEP delivery sites (2018 USD). Costs are presented in 2018 prices and inflated to 2021 prices. The annual cost/PrEP client varied by service delivery model, from $394 (AGYW) to $655 (integrated model). Cost differences were driven largely by client volume, which impacted the relative costs of program support and technical assistance assigned to each PrEP client. Direct service delivery costs ranged narrowly from $205-212/PrEP-client and were a key component in the cost of PrEP, representing 35–65% of total costs. The results show that, even when integrated into full service delivery models, accessing vulnerable, marginalised populations at substantial risk of HIV infection is likely to cost more than previously estimated due to the programmatic costs involved in community sensitization and client support. Improved data on individual client resource usage and outcomes is required to get a better understanding of the true resource utilization, expected outcomes and annual costs of different PrEP service delivery programs in Zambia.

## Introduction

Following several clinical trials and observational studies [1–3], the World Health Organization (WHO) has recommended that pre-exposure prophylaxis (PrEP) be offered to anyone at substantial risk of HIV infection [4]. Several low- and middle-income countries (LMICs) have adopted these guidelines and are currently implementing national PrEP programs [5]. However, limited data are available to inform the total cost and affordability of these programs, which is crucial for policy decisions around implementation strategies for HIV prevention programs.

A recent systematic review of modelling studies looking at the impact, cost and cost-effectiveness of PrEP in SSA reported that PrEP can be cost-effective as an addition to HIV prevention packages in some cases, but that is not cost-effective to introduce PrEP prior to the expansion of other prevention interventions [6]. Most of the included studies largely focused on direct service delivery costs and many do not take into account routine PrEP program support costs. Case et al. (2019) call for additional modelling that includes updated programmatic information and real-world resource utilization data to inform efficient use of resources [6]. The costs of PrEP programs implemented in routine care settings in sub-Saharan Africa (SSA) are largely unreported and those that are, have thus far focused primarily on targeting key populations including female sex workers (FSW), men who have sex with men (MSM), and priority populations such as adolescent girls and young women (AGYW). Evidence demonstrating the variations in costs of these different PrEP programs, service delivery strategies, and resource utilization in SSA is limited, with only a few demonstration projects having published the costs of providing PrEP in these settings [7–12].

Estimating the costs of scaling up PrEP programs is complicated by the changing ‘population-at-risk’ for any given time period. Unlike ART, which is intended as a life-long treatment program under which optimal treatment requires 100% adherence, PrEP is effective when taken during periods of high risk, meaning that cycling on and off PrEP may be appropriate for effective use [13]. This complicates analyses of budgetary needs and scale-up costs of PrEP programs as it is unclear what patterns of PrEP persistence should and will look like, and how this will affect the long-term costs of PrEP service delivery and program support. Zambia is has been facing with national PrEP implementation and scale-up.

Zambia, a lower-middle income country in southern Africa with an estimated 48,000 new HIV infections in 2018, is investing in scaling up its PrEP program [14]. Zambia has adopted daily oral PrEP containing tenofovir and emtricitabine as an additional HIV prevention strategy, recommending PrEP for all HIV-negative persons at high risk of HIV acquisition [15]. By September 2018, PrEP was offered in 162 sites across nine of the ten provinces, with 3,626 people at risk of HIV infection initiated on PrEP; one year later, this had increased to 23,327 at 728 sites across all provinces [16]. These were largely serodiscordant couples, but also included AGYW ages 15–24 (35%), FSW (9%), and MSM (3%) [17]. The Zambian Ministry of Health aims to further expand PrEP services, but has limited information on the cost and budgetary impact of this national scale-up.

We present the results of a costing study of PrEP implementation in Zambia, aiming to provide cost estimates of PrEP provision by different service delivery models aiming to reach different at-risk populations. We also present costs per PrEP-month of effective use, using aggregate PrEP persistence data, and compare that to costs for perfect use.

## Methods

### Study setting

Zambia’s PrEP guidelines stipulate that those eligible for PrEP and identified as being at substantial risk of HIV infection (as defined as engaging in one or more risky activities in the past six months) be offered PrEP (Table 1). We costed five service delivery models in Zambia that offered PrEP to specific at-risk populations: AGYW (AGYW-focused), FSW and MSM (MSM/FSW-focused), and anyone at risk of HIV infection visiting a primary healthcare facility (Integrated 1–3). The AGYW-focused, MSM/FSW-focused and Integrated 1 models were run by one implementation partner, while the other two integrated models were each run by different implementation partners. These models are described in detail in Table 2.

**Table 1:** PrEP clinical activities adapted from the Zambian PrEP implementation framework [18].

	Year 1 (at least 6 visits)						Year 2 (at least 4 visits)			
	Initial visit and PrEP initiation	Follow-up months								
		1	3	6	9	12	15	18	21	24
Confirmation of HIV-negative status	x	x	x	x	x	x	x	x	x	x
Serum Creatinine	x	x	x	x	x	x				x
Hepatitis B surface antigen	x			x						
Hepatitis C antibody*	x			x						
Pregnancy testing**	x									
Rapid Plasma Reagin (RPR)	x			x				x		
Other screening for sexually transmitted infections (STIs)	x		x	x	x	x	x	x	x	x
Alanine Transaminase (ALT)	x			x						x

*Included for men-who-have-sex-with-men (MSM) only

**Included for women only

Abbreviations. PrEP: Pre-exposure prophylaxis; HIV: Human Immunodeficiency Virus; RPR: Rapid Plasma Reagin; STI: Sexually transmitted infection; ALT: Alanine Transaminase

**Table 2:** Description of the service delivery models included.

Service delivery model	AGYW-focused	MSM/FSW-focused	Integrated 1	Integrated 2	Integrated 3
Summary	Fixed facilities and community outreach	Tailored and targeted community-based interventions	PrEP services integrated into HIV testing and counselling services	PrEP services integrated into HIV testing and counselling services	PrEP services integrated into HIV testing and counselling services
Population targeted	AGYW	FSW and MSM	All at risk of HIV-infection	All at risk of HIV-infection	All at risk of HIV-infection
Model details	A broad, multi-component package of evidence-based health, educational and social interventions aimed at reducing new HIV infections. The AGYW-focused program was integrated as part of the DREAMS program for adolescent girls and young women [19].	Targeted community-based demand creation programs with referrals to local facilities for PrEP initiation and follow up.	Program that focused on health promotion activities for facility personnel and service provider training and sensitisation that focused on improving client-provider interactions. Community Health Workers referred community members to local facilities for PrEP initiation and follow up.	Focused resources on site readiness through preliminary site assessments, training and community consultations. Additionally, there were several international technical support visits from implementation partners.	The third model was based on the Community HIV Epidemic Model (CHEC) of care [20], with costs incurred for community education, mobilization, and PrEP sensitization alongside training of health care workers (HCWs) on KP sensitivity and PrEP service delivery.
Management type	NGO	NGO	NGO	NGO	NGO
Location of partner opeations	Lusaka and Copperbelt provinces	Central, Copperbelt, Lusaka, North-Western and Southern provinces	Copperbelt province	Northern and Muchinga provinces	Lusaka and Central provinces
Demand creation and community sensitisation activities	Community sensitization and demand creation activities through short-term community mobilisers	Short-term mobilisers and peer navigators spent time in the local communities to specifically reach FSW and MSM populations, create demand for PrEP refer them for services at the clinics	Trained community health workers to sensitize the community about PrEP and refer those interested in PrEP to the local clinic	Community consultations took place	Community consultations, PrEP orientation sessions and key population sensitization activities took place
PrEP initiation and follow-up location	Specialised DREAMS centers with an integrated community health worker who was trained on PrEP provision	Government-run primary healthcare centers	Government-run primary healthcare centers	Government-run primary healthcare centers	Government-run primary healthcare centers
PrEP-client support	Continued PrEP-client support using peer navigators	Continued PrEP-client support through mobilisers and peer navigators	Continued PrEP-client support using CHWs	Technical support provided to clinic and facility staff to improve PrEP-client support through the facilities	Continued PrEP-client support using CHWs
Number of sites in 2018	13	4	90	4	27
Total 2018 PrEP volume	908	213	322	214	148
Total target 2019 PrEP volume	1408	500	2133	435	Unknown

* DREAMS (Determined, Resilient, Empowered, AIDS-free, Mentored and Safe) partnership is a public-private partnership aimed at reducing rates of HIV among AGYW in highest HIV burden countries. It is largely funded and implemented by the United States Agency for International Development (USAID).

** CHEC (Community HIV Epidemic Model) is an “innovative differentiated service delivery (DSD) model for human immunodeficiency virus (HIV)/ acquired immunodeficiency syndrome (AIDS) care in Zambia” being implemented by The Center for International Health, Education, and Biosecurity (CIHEB) of the Institute of Human Virology, at the University of Maryland, Baltimore (UMB). This model “addresses all three of the United Nations Program on HIV/AIDS (UNAIDS) 90-90-90 targets and utilizes a peer-to-peer approach to conduct health education, provide targeted HIV testing services (HTS) in the community, refer and link HIV-infected clients to treatment services, as well as HIV-uninfected clients to preventative health services, and to deliver antiretrovirals (ARVs) to eligible people living with HIV (PLHIV) in the community to ensure adherence and sustain viral suppression.”

### Costs

The costs of the PrEP programs were estimated from the providers’ perspective, with data collected in 2018 and reported in 2018 US dollars (USD). Costs were also inflated to 2021 USD using a cumulative inflation factor of 1.36 and are presented in S1 Table [22]. Costs collected in Zambian Kwacha were converted to the USD equivalent based on the average exchange rate for 2018 of 10.4988 ZMW to 1 USD (January-December) [23]. We used expenditure analysis in estimating financial costs and tracked actual implementation expenses including training, demand creation and technical support. At the facility-level we only included costs directly related to PrEP programming such as counsellor-time, facility space and equipment. We categorized the costs into program costs and direct service delivery costs. Program costs included project start-up costs, training, sensitization as well as provider and client support costs. Direct service delivery costs included clinical personnel, supplies, laboratory tests and drugs (Table 3).

**Table 3:** Cost methods and unit costs for direct service delivery costing.

Resource	Method for estimating cost	Item	Unit	Cost (ZMW)	Cost (USD)	Source
Drugs	Drug costs were calculated by the drugs prescribed, the dosage required and the duration the drug would be taken for (in this case, 12 months). According to guidelines, PrEP-clients are required to pick up monthly drug supplies for the first twelve months. The published national drug unit costs were applied to determine total drug cost per client.	Emtricitabine + Tenofovir	Tablet	2.51	0.22	Medical Stores Limited (MSL) Catalogue 2016 [prices unchanged 2016–2018]
Laboratory tests	The number of tests performed per PrEP-client for the study period was extrapolated based the Zambian guidelines and target population type. According to the Zambia guidelines, all PrEP-clients undergo the same monitoring labs, with the exception of the addition of pregnancy testing for women and Hepatitis C antibody testing for MSM; for the purposes of this analysis, we assumed that all clients received all required lab tests at each visit. Since the cost of public sector laboratory tests are not available in Zambia, we estimated public sector laboratory costs by adjusting the 2018 South African National Health Laboratory Services pricelists. Using the published cost of an HIV viral load test performed at a central government laboratory in Zambia, we calculated adjusted prices for the applicable laboratory tests for PrEP in Zambia.	Creatinine	Test	24.07	2.29	South Africa’s 2018 NHLS pricelist, adjusted using ratio of South Africa public sector viral load price to Zambia’s public sector viral load price as reported in Nichols et al. 2020 [21] (no public laboratory price list available for general non-viral load tests)
		ALT	Test	36.03	3.43	
		HBsAg	Test	99.85	9.51	
		RPR/RST	Test	15.59	1.49	
		Pregnancy*	Test	67.33	6.41	
		STIs	Test	55.85	5.32	
		Hep C antibody**	Test	99.85	9.51	
Clinic visit costs***	We costed three different types of visits: initiation, monitoring/follow-up and drug pick-up visits. These PrEP clinic visit costs were based on micro-costing at two PrEP delivery sites in Lusaka and include staff time, consumables (including HIV test and condoms), and overheads (equipment, space, water, effluent, and electricity). Staff time required for each visit type by staff cadre was estimated using information provided by healthcare workers and implementing partners. The staff cost per minute was calculated using Ministry of Health (MOH) salaries for each respective cadre of staff involved in PrEP delivery. Clinical staff salaries were obtained from the published list of public sector salaries for that cadre. We estimated the consumables, such as needles and gloves, used per visit type through discussion with the staff and mapping what activities occur during a visit at the two clinics in Lusaka. Unit costs of consumables were obtained from supplier price lists (MSL catalogue, 2016 USD [prices unchanged 2016–2018]). Equipment and overhead costs were estimated across ten healthcare facilities in two provinces (Central and Copperbelt) through a separate project aimed at costing standard ART visits [31]. These costs then applied to our study as ART patients frequent the same spaces and spent similar amount of time in a facility as with PrEP visits.	PrEP initiation visit	Visit	42.36	4.03	MSL catalogue, 2016 USD [prices unchanged 2016–2018] Zambia MOH salary scales
		PrEP follow-up visit at month 1	Visit	39.57	3.77	
		PrEP follow-up visits at months 3, 9 and 12	Visit	40.37	3.84	
		PrEP follow-up visit at month 6	Visit	41.96	4.00	
		Pharmacy pick-up	Visit	6.19	0.59	

*For women only

**For men-who-have-sex-with-men only

***Visits include staff time, consumables, and overheads (equipment, shared space, dedicated space and facility-level overheads). Consumables differ by visit as per guidelines. Consumables include the HIV test and condoms as appropriate.

Abbreviations. ZMW: Zambian Kwacha; USD: United States Dollar; MSL: Medical Stores Limited; PrEP: Pre-exposure prophylaxis; HIV: Human Immunodeficiency Virus; NHLS: National Health Laboratory Service; RPR: Rapid Plasma Reagin; RST: Rapid Syphilis Test; STI: Sexually transmitted infection; ALT: Alanine Transaminase; HBsAg: Hepatitis B surface antigen; MOH: Ministry of Health; MSM: Men-who-have-sex-with-men.

Annualized program start-up, provider, and client support costs were based on program expenditure data and the number of PrEP sites and clients in 2019. Program start-up costs included training (annualized over two years), initial client demand creation activities and communication. Program support costs included recurring costs such as ongoing technical assistance and patient support costs (for example, peer navigators, peer support groups, social media support). We verified costing data via interviews with clinical staff and implementing partners and triangulation with program records. We analyzed costs in Excel 2018 (Microsoft, Redmond, USA). Ethical approval, as well as a consent process, were not required for this study as this was not human subjects’ research, given that only aggregate program data were used.

### Analysis

We estimated the annual cost of providing PrEP services assuming fidelity to the expanded 2018 Zambian PrEP guidelines for 12-months of PrEP (Table 1). We estimated patient resource use based on these guidelines from the day of PrEP initiation for 12 months, assuming continuous PrEP use for the duration. These direct service delivery costs included drugs, laboratory tests, and clinic visits. Assuming full adherence to guidelines, we then applied the unit costs to the resources expected to be used over the 12-month period by sex and risk group (Table 3).

Furthermore, we modelled the cost of PrEP provision for a hypothetical cohort of 1000 individuals taking PrEP over a 12-month period under two scenarios: 1) all PrEP-clients took PrEP continuously for 12-months and 2) using the actual persistence rates from programmatic PrEP data for visits at months 1, 3, 6, 9 and 12 for heterosexual men, women and MSM. Using the population-specific service delivery costs described above, we determined a total cost per month based on these persistence rates. We then calculated the cumulative number of protected months for each visit over a 12-month period and, using this, calculated the cost of PrEP per person-month effectively covered on PrEP.

## Results

The number of PrEP-clients and sites varied across service delivery models, with the average number of PrEP-clients per site ranging from 3.6 in the Integrated 1 models and almost 70 in the AGYW model (Table 4). Start-up costs per site ranged from just over $400 for the Integrated 3 model to over $5000 for the AGYW program. However, this cost difference between the two models disappeared when considering the number of PrEP-clients per site, with both models costing $73 per PrEP-client per site. The Integrated 1 model incurred the largest start-up costs per PrEP-client per site at $230, while the Integrated 2 program was the least costly at $26 per PrEP-client per site.

**Table 4:** 2018 costs of PrEP-provision according to Zambian guidelines (USD).

Program Type	AGYW	FSW/MSM	Integrated 1	Integrated 2	Integrated 3
**Number of PrEP clients initiated in program**	908	213	322	214	148
**Number of total sites**	13	4	90	4	27
**Average number of PrEP-clients/site**	69.8	53.3	3.6	53.5	5.5
**Start-up costs/site**					
Community sensitization/consultation	0	0	156	654	242
Training	3979	2844	668	308	160
Site assessments	0	0	0	422	0
Equipment	1149	1149	0	0	0
**Average cost of start-up/site**	**5128**	**3992**	**824**	**1384**	**403**
**Average cost of start-up/site/PrEP-client**	**73**	**75**	**230**	**26**	**73**
**Recurrent costs**					
**Program support costs/PrEP-client**					
Technical support	0	0	0	154	265
Training	15	19	82	17	26
PrEP-client support	63	83	0	0	85
PrEP provider support	13	0	128	0	0
Demand creation communication	22	31	2	0	0
**Average program support costs/PrEP-client (% of recurrent costs)**	**113 (35%)**	**133 (39%)**	**212 (51%)**	**171 (45%)**	**377 (65%)**
**Direct service delivery costs/PrEP-client**					
Staff & Overheads	46	46	46	46	46
Labs and monitoring	64	68	62	62	62
Consumables	13	13	13	13	13
PrEP Drugs	86	86	86	86	86
**Average direct service delivery costs/PrEP-client (% of recurrent costs)**	**208 (65%)**	**212 (61%)**	**205 (49%)**	**205 (55%)**	**205 (35%)**
**Total recurrent cost/PrEP-client/year**	**320**	**345**	**418**	**377**	**582**
**Total average cost/PrEP-client/year**	**394**	**420**	**648**	**403**	**655**
Total cost/person-month*	**33**	**35**	**54**	**34**	**55**

**Assuming 12 months effective PrEP coverage per PrEP-clientAbbreviations. PrEP: Pre-exposure Prophylaxis; USD: United States Dollar; AGYW: Adolescent girls and young women; MSM: Men-who-have-sex-with-men; FSW: Female Sex Worker.

**Fig 1:**
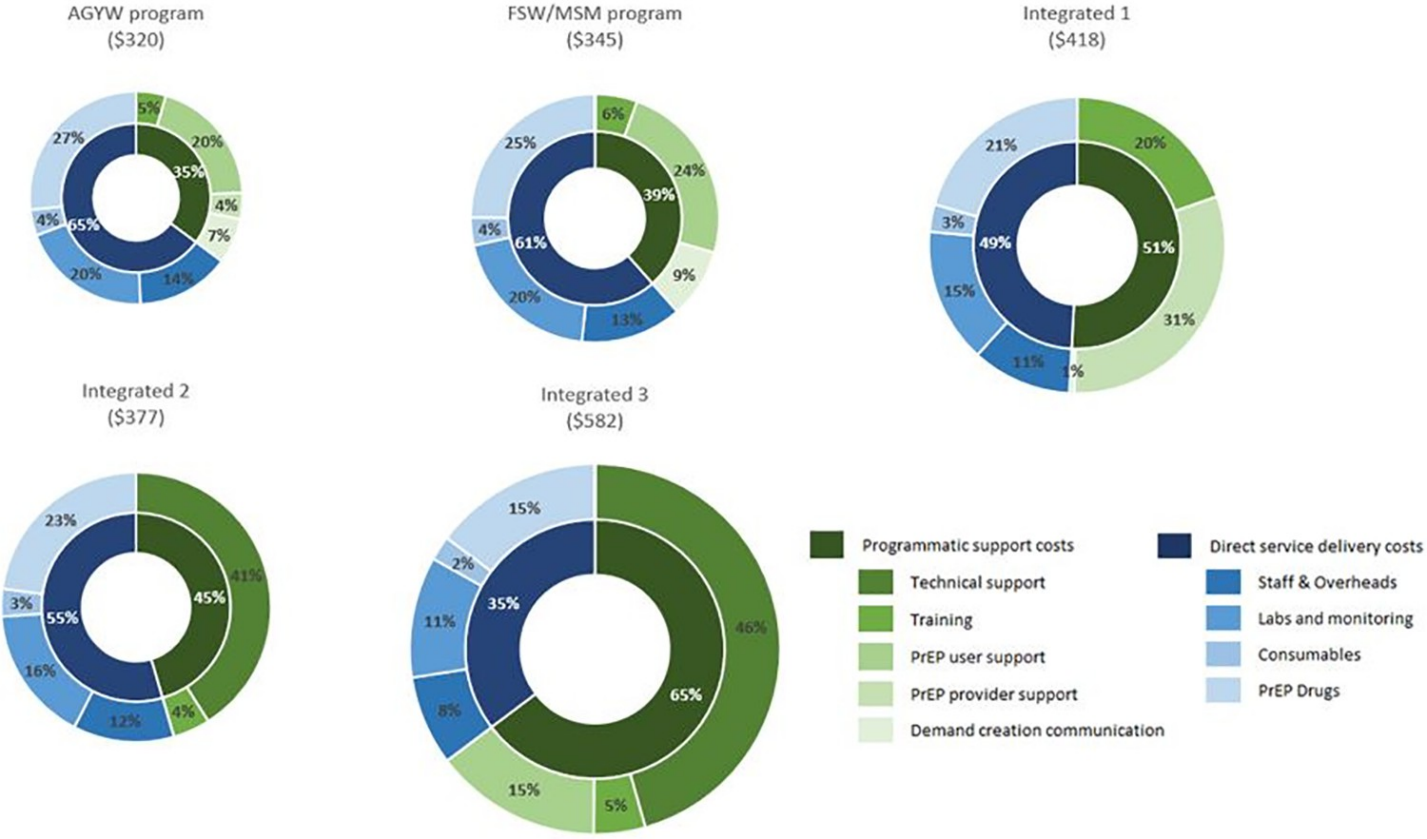
Proportion of total annual recurrent costs per PrEP-client by cost inputs (2018 USD)*. *Figures are approximately sized to illustrate the overall annual cost per PrEP-client (i.e. the larger the figure, the costlier the program per PrEP-client). Costs have been inflated to 2021 from their 2018 cost prices.

Recurrent costs per PrEP-client also varied by program, driven by the differences in programmatic support costs. These ranged from $113 for the AGYW program to $377 for the third integrated program. Direct service delivery costs did not vary greatly, with the variation due to the different monitoring laboratory tests guidelines require for each target population. The proportion of recurrent costs attributable to programmatic support costs ranged from just over a third (35%) for the AGYW-focused program to almost two-thirds for the third integrated program (65%), with the two priority population focused programs spending a lower proportion of these costs on programmatic support costs than the integrated programs (Fig 1). The drug costs carried the highest proportion of the service delivery costs at about 40%, followed by monitoring labs at about a third of the cost. However, when compared to the costs of drugs and labs as a proportion of the entire recurrent program costs, this fell to between 15–27% and 11–21% respectively.

When taking the average number of PrEP-clients initiated per site into account, and combining program start-up and recurrent support costs and assuming 12 months of continuous PrEP-use, the annual cost per PrEP initiate varied greatly by program type, with two of the integrated programs being most costly at $648 and $655 per PrEP-initiate. The FSW/MSM-focused program and second integrated program cost $420 and $403 per PrEP initiate respectively. The least costly program per PrEP initiate, considering start-up costs, number of sites and site volume was the AGYW-focused program at $394 per PrEP initiate. These cost differences were driven largely by the differences in the average number of PrEP-clients initiated per site across each program. The two integrated models (1 and 3) cost $54 and $55 per PrEP-client per person-month respectively, assuming 12 months of continuous PrEP use, almost double that of the AGYW-focused program at $33.

**Fig 2:**
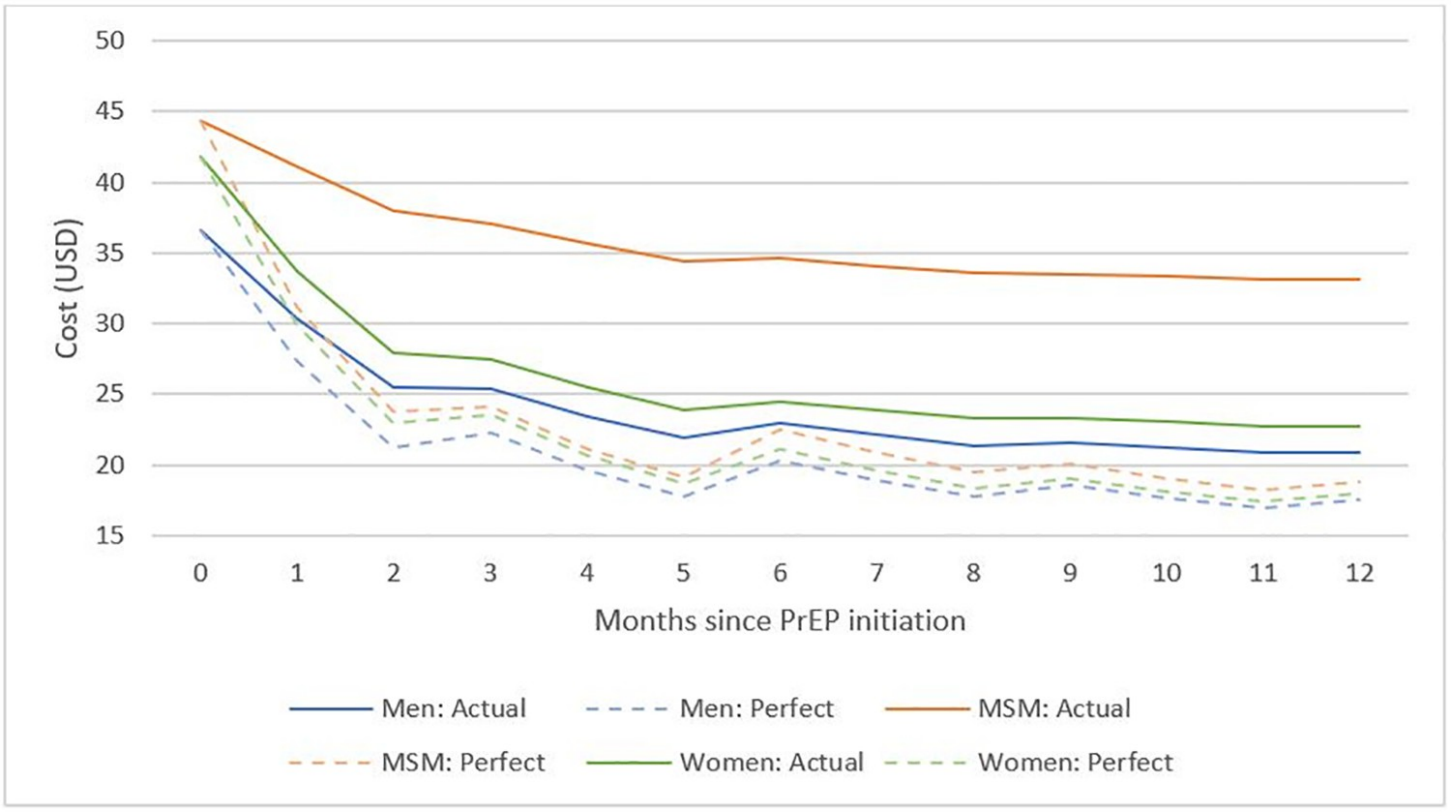
Sensitivity analysis of cost of PrEP provision per person-month effectively covered on PrEP, using aggregate persistence data*. *Persistence was reported for months 1, 3, 6, 9 and 12—for the remainder of the months, and average was taken using the adjacent two data points (i.e. for month 2 persistence, an average was taken of the reported persistence for month l and month 3. Costs are reported in 2021 USD and only include direct service delivery costs.

Comparing two simulation scenarios, one with perfect PrEP persistence and the other using PrEP continuation data from Zambia, we see that the cost of PrEP provision per person-month effectively covered on PrEP decreases over time for all groups under both scenarios (Fig 2). However, this decrease is less substantial when there is imperfect persistence. For example, the cost of PrEP provision per person month among MSM is 76% greater at 12 months when using actual data on persistence in that risk group as compared to assuming perfect persistence. For women, this difference is smaller at 27% ($20 per person at 12 months with actual persistence rates versus $16 for perfect persistence). The smallest difference is among men with a 19% greater cost of PrEP provision per person month at 12 months.

## Discussion

Modelling studies for PrEP in SSA indicate that PrEP can be cost effective when targeted to specific high-risk groups [6]; however, most models have not used data from real-world implementation settings. To date only a few studies have reported the cost of providing PrEP to priority populations in SSA using data from demonstration projects or routine implementation. In this study, we address this gap and add to the small but growing PrEP cost literature by describing the guideline-based costs of PrEP service delivery in the context of a generalized epidemic in an LMIC. We estimated that the overall average cost to provide PrEP to an at-risk individual for a full 12 months in Zambia ranged from $394 to $655, or $33 to $55 per person-month on PrEP. This varied by the different service delivery models, with programs focused on reaching priority populations (AGYW, FSW/MSM) generally costing less than those integrated into existing healthcare facilities, when taking into consideration the average number of PrEP-clients per site. The differences in costs between the service delivery models are largely driven by differences in program support with programs relying heavily on provider training and technical support (the integrated programs) costing more than those focused on community outreach and PrEP-client support (the population-specific programs). Variation in costs between the three integrated models were driven by PrEP-client volumes per site, with the models with higher client volumes per site demonstrating lower costs per client on PrEP than those with low client volumes. These costs are based solely on the Zambian PrEP policy, which assumes that all patients were treated precisely according to these guidelines, with no variation among patients based on condition, logistical challenges, or other potential variants.

The costs of PrEP provision we report in this study, ranging from $394 to $655 are generally higher than those documented in other costing studies from the region. In Kenya and Uganda, the incremental cost of providing PrEP to serodiscordant couples under study settings was just over $305 and $408 respectively [8, 10], while in South Africa PrEP provision cost about $127 per year for FSW [7]. A study in Zimbabwe reported the cost per PrEP client initiated as $238 while another study in Kenya reported an overall cost of $256 [11, 24]. Compared to our calculated costs per person-month, ranging from $37 to $61, other studies were also lower at $26.52 under study settings or $16.54 under MOH scenario and $21.32 and $28.92 study, $14.52 for adolescent girls and young women [12, 25, 26]. One possible reason for these differing results are the relatively low PrEP client volumes in these programs compared to those reported in other studies, which ranged from 219 to almost 5000. In Kenya, authors report that the cost per client-month of PrEP dispensed is reduced substantially if PrEP delivery is scaled up. This is supported in other HIV scale-up studies that have demonstrated that early implementation costs are greater per patient served (“U-shaped incremental cost curve”) [27–29]. Understanding where we are on the U-shaped curve will assist with planning and to ensure programs are adequately resourced. Similar to HIV treatment programs focusing on hard-to-reach key populations, targeted PrEP programs require substantial investment in order to establish and maintain successful programs [30–33]. More intensive programmatic inputs and larger PrEP-client volumes are required per site for the key population-focused programs to reach the minimum cost/client; however, this investment may be necessary to effectively reach these groups, especially as more than half of all new HIV infections were among key populations in 2018, despite being a minority of the overall population [34].

It should also be noted that the start-up cost of scaling up any of these programs nationally likely exceeds the simple multiplication of start-up costs and number of sites. Particularly for the integrated approach, many of the sites that have been reached to date have been relatively easy to reach. The cost to scale-up to all facilities may therefore start to increase once smaller health facilities that serve fewer patients are reached (the other side of the U-shaped incremental cost curve), with additional travel and staff costs to get these sites started [29]. There are now more than 1500 health facilities in Zambia that provide ART services (e.g. these could potentially also all provide PrEP services), of which 24% provide service to 80% of patients, so an effort to scale-up PrEP to all facilities may be cost-prohibitive.

Using PrEP persistence data from Zambia to simulate the cost of PrEP provision over time, we observe that low persistence results in higher costs per PrEP-client, up to 76%. Therefore, understanding PrEP persistence in the population is key to planning and budgeting. Our data show that it is costly to initiate someone on PrEP who only persists on PrEP for a few months. This does not necessarily mean that a PrEP program is not cost-effective; it just means that this low-usage, or in-and-out cycling, needs to be accounted for when planning PrEP provision and program scale-up. These costs vary with the duration of at-risk periods when PrEP is required–longer periods on PrEP require more visits and medications resulting in high costs of continual PrEP use per year per PrEP-client, though lower per month costs. Preliminary research has shown that, despite heterogeneity within sub-groups of PrEP clients, PrEP persistence in Zambia is low, with retention at three months only 27% in one study [16, 17]. This means that the cost per initiate will likely decline rapidly for a given PrEP initiation cohort. Furthermore, the cost per PrEP initiate may be lower depending on how many carry-over 2018 PrEP clients there were in 2019.

This analysis is subject to a number of limitations. First, we relied on implementing partners and provider reports and some resource use may not have been recorded. We were unable to directly observe time spent for PrEP alone from staff time on other primary care and relied on provider recall and estimates to determine time per visit. This may mean our staff cost estimates are lower than would be expected in a low HIV prevalence setting or where care is not integrated. We also excluded salary costs of ancillary implementing partner staff who were involved in PrEP-program planning as they were reported inconsistently across partners. Including these costs would further increase this cost per PrEP client served. These lowest cost thresholds will not apply to truly integrated models where capacity and resources are shared. Furthermore, we did not have the individual site-level costs within each service delivery model so the ‘per site’ costs reported are an equal division of costs across all sites. As such, we are unable to report variation summary statistics and acknowledge that this likely over-estimates or under-estimates the site-level costs on an individual site level. Additionally, this analysis does not investigate the effect that changes in integration and standardization would have on the cost of PrEP provision across different clinic sizes. This is an important factor when thinking about PrEP provision to small or more rural clinics where high PrEP-client volumes may not be achievable. Ensuring access, however, even in remote or low-volume facilities is important to ensure equitable PrEP access, and costs associated with a lower PrEP client volume should be planned for. As this analysis is based on national guidelines and does not include patient or program outcomes, we are not making any conclusions comparing the effectiveness of the various models, but are providing budgetary information only. Further costing work is needed to elucidate the effect of PrEP persistence on the overall costs of PrEP provision. Despite these limitations, this study provides a robust estimate of the costs of PrEP provision under three different service delivery models intended to guide future scale-up. This information will support budgeting and financial planning for PrEP services as Zambia scales up HIV prevention and access to services to achieve national and international targets.

## Conclusions

Given finite resources, methods to ensure low incremental costs for PrEP provision are important. This work demonstrates that the routine implementation and service delivery of PrEP may be more costly than modelling and early demonstration projects indicated. In addition to direct delivery cost, PrEP programs incur substantial costs for demand creation and PrEP-client support. The costs involved in these activities to reach key and priority populations should not be discounted when budgeting for PrEP program scale-up.

## Supporting information

S1 Table 2021 Inflated costs of PrEP-provision according to Zambian guidelines (USD). (DOCX)

## References

[1] Fonner VA, Dalglish SL, Kennedy CE, Baggaley R, O’Reilly KR, Koechlin FM, et al. Effectiveness and safety of oral HIV preexposure prophylaxis for all populations. Aids. 2016;30(12):1973–83. doi: 10.1097/QAD.0000000000001145 27149090 PMC4949005

[2] Baeten JM, Donnell D, Mugo NR, Ndase P, Thomas KK, Campbell JD, et al. Single-agent tenofovir versus combination emtricitabine plus tenofovir for pre-exposure prophylaxis for HIV-1 acquisition: an update of data from a randomised, double-blind, phase 3 trial. The Lancet Infectious diseases. 2014;14(11):1055–64. doi: 10.1016/S1473-3099(14)70937-5 25300863 PMC4252589

[3] Wilton J, Senn H, Sharma M, Tan DH. Pre-exposure prophylaxis for sexually-acquired HIV risk management: a review. HIV AIDS (Auckl). 2015;7:125–36. doi: 10.2147/HIV.S50025 25987851 PMC4422285

[4] WHO. Policy brief: pre-exposure prophylaxis (PrEP): WHO expands recommendation on oral pre-exposure prophylaxis of HIV infection (PrEP). Geneva: World Health Organization; 2015 2015. Contract No.: WHO/HIV/2015.48.

[5] PrEPWatch. Where do you fit on the map? [https://www.prepwatch.org/.]

[6] Case KK, Gomez GB, Hallett TB. The impact, cost and cost-effectiveness of oral pre-exposure prophylaxis in sub-Saharan Africa: a scoping review of modelling contributions and way forward. Journal of the International AIDS Society. 2019;22(9):e25390. doi: 10.1002/jia2.25390 31538407 PMC6753289

[7] Eakle R, Gomez GB, Naicker N, Bothma R, Mbogua J, Cabrera Escobar MA, et al. HIV pre-exposure prophylaxis and early antiretroviral treatment among female sex workers in South Africa: Results from a prospective observational demonstration project. PLoS medicine. 2017;14(11):e1002444. doi: 10.1371/journal.pmed.1002444 29161256 PMC5697804

[8] Irungu EM, Sharma M, Maronga C, Mugo N, Ngure K, Celum C, et al. The Incremental Cost of Delivering PrEP as a Bridge to ART for HIV Serodiscordant Couples in Public HIV Care Clinics in Kenya. AIDS research and treatment. 2019;2019:4170615. doi: 10.1155/2019/4170615 31186955 PMC6521338

[9] Roberts DA, Barnabas RV, Abuna F, Lagat H, Kinuthia J, Pintye J, et al. The role of costing in the introduction and scale-up of HIV pre-exposure prophylaxis: evidence from integrating PrEP into routine maternal and child health and family planning clinics in western Kenya. Journal of the International AIDS Society. 2019;22 Suppl 4:e25296. doi: 10.1002/jia2.25296 31328443 PMC6643078

[10] Ying R, Sharma M, Heffron R, Celum CL, Baeten JM, Katabira E, et al. Cost-effectiveness of pre-exposure prophylaxis targeted to high-risk serodiscordant couples as a bridge to sustained ART use in Kampala, Uganda. Journal of the International AIDS Society. 2015;18(4 Suppl 3):20013. doi: 10.7448/IAS.18.4.20013 26198348 PMC4509901

[11] Mangenah C, Nhamo D, Gudukeya S, Gwavava E, Gavi C, Chiwawa P, et al. Efficiency in PrEP Delivery: Estimating the Annual Costs of Oral PrEP in Zimbabwe. AIDS Behav. 2022 Jan;26(1):161–170. doi: 10.1007/s10461-021-03367-w 34453240 PMC8786759

[12] Wanga V, Peebles K, Obiero A, Mogaka F, Omollo V, Odoyo JB, et al. Cost of pre-exposure prophylaxis delivery in family planning clinics to prevent HIV acquisition among adolescent girls and young women in Kisumu, Kenya. PLoS One. 2021;16(4):e0249625. doi: 10.1371/journal.pone.0249625 33857195 PMC8049260

[13] Bekker LG, Rebe K, Venter F, Maartens G, Moorhouse M, Conradie F, et al. Southern African guidelines on the safe use of pre-exposure prophylaxis in persons at risk of acquiring HIV-1 infection. Southern African journal of HIV medicine. 2016;17(1):455. doi: 10.4102/sajhivmed.v17i1.455 29568613 PMC5843155

[14] UNAIDS. Country: Zambia 2016 [https://www.unaids.org/en/regionscountries/countries/zambia].

[15] Republic of Zambia Ministry of Health. Zambia Consolidated Guidelines for Treatment & Prevention of HIV Infection. 2018. [https://www.prepwatch.org/wp-content/uploads/2018/12/Zambia-Consolidated-Guidelines2018.pdf].

[16] Claassen CW, Mumba D, Njelesani M, Nyimbili D, Mwango LK, Mwitumwa M, et al. Initial implementation of PrEP in Zambia: health policy development and service delivery scale-up. BMJ open. 2021;11(7):e047017. doi: 10.1136/bmjopen-2020-047017 34244265 PMC8273462

[17] Claassen C, Mumba D, Njelesani M, Nyimbili D, Mwango L, Mubanga E, et al., editors. First year of implementation of PrEP in Zambia: Service delivery roll-out and scale-up. IAS 2019; 2019; Mexico City, Mexico.

[18] Republic of Zambia Ministry of Health and National HIV/AIDS/STI/TB Council. Implementation Framework & Guidance For Pre-exposure Prophylaxis of HIV Infection. 2018.

[19] USAID. DREAMS: PARTNERSHIP TO REDUCE HIV/AIDS IN ADOLESCENT GIRLS AND YOUNG WOMEN 2021 https://www.usaid.gov/global-health/health-areas/hiv-and-aids/technical-areas/dreams.

[20] Center for International Health Education and Biosecurity, Institute of Human Virology, University of Maryland School of Medicine. The UMB Community HIV Epidemic Model Differentiated Prevention, Testing, and ART Delivery for the General Population and Pregnant & Breast-Feeding Women and their Children in Zambia http://ciheb.org/media/SOM/Microsites/CIHEB/documents/5-Zambia---UMB-CIHEB-(2).pdf.

[21] Nichols BE, Girdwood SJ, Shibemba A, Sikota S, Gill CJ, Mwananyanda L, et al. Cost and Impact of Dried Blood Spot Versus Plasma Separation Card for Scale-up of Viral Load Testing in Resource-limited Settings. Clinical infectious diseases: an official publication of the Infectious Diseases Society of America. 2020;70(6):1014–20. doi: 10.1093/cid/ciz338 31321438 PMC7931834

[22] World Bank. Inflation, consumer prices (annual %)—Zambia https://data.worldbank.org/indicator/FP.CPI.TOTL.ZG?locations=ZM.

[23] Bank of Zambia. Bank of Zambia Average Exchange Rates 2018 https://www.boz.zm/average-exchange-rates.htm.

[24] Mutuku U, Forsythe, S., Glaubius, R., Were, D., Musau, A. Estimating the Costs of PrE-exposure Prophylaxis (PrEP) in Ten Counties of Kenya. International Conference for AIDS and STIs in Africa 2019.

[25] Peebles K, Mugwanya KK, Irungu E, Odoyo J, Wamoni E, Morton JF, et al. Low costs and opportunities for efficiency: a cost analysis of the first year of programmatic PrEP delivery in Kenya’s public sector. BMC health services research. 2021;21(1):823. doi: 10.1186/s12913-021-06832-3 34399736 PMC8365926

[26] Roberts A, Barnabas, RV., Abuna, F., Lagat, H., Kinuthia, J., Pintye, J., et al. The Cost of PrEP Delivery in Kenyan Antenatal, Postnatal, and Family Planning Clinics. Conference on Retroviruses and Opportunistic Infections; March 4–7 2019; Seattle, WA, USA2019.

[27] Guinness L, Kumaranayake L, Rajaraman B, Sankaranarayanan G, Vannela G, Raghupathi P, et al. Does scale matter? The costs of HIV-prevention interventions for commercial sex workers in India. Bulletin of the World Health Organization. 2005;83(10):747–55. doi: /S0042-96862005001000011 16283051 PMC1852061

[28] Kumaranayake L. The economics of scaling up: cost estimation for HIV/AIDS interventions. Aids. 2008;22 Suppl 1:S23–33. doi: 10.1097/01.aids.0000327620.47103.1d 18664950

[29] Nichols BE, Girdwood SJ, Crompton T, Stewart-Isherwood L, Berrie L, Chimhamhiwa D, et al. Monitoring viral load for the last mile: what will it cost? J Int AIDS Soc. 2019;22(9):e25337. doi: 10.1002/jia2.25337 31515967 PMC6742838

[30] Cham HJ, MacKellar D, Maruyama H, Rwabiyago OE, Msumi O, Steiner C, et al. Methods, outcomes, and costs of a 2.5 year comprehensive facility-and community-based HIV testing intervention in Bukoba Municipal Council, Tanzania, 2014–2017. PLoS One. 2019;14(5):e0215654. doi: 10.1371/journal.pone.0215654 31048912 PMC6497243

[31] Meyer-Rath G, van Rensburg C, Chiu C, Leuner R, Jamieson L, Cohen S. The per-patient costs of HIV services in South Africa: Systematic review and application in the South African HIV Investment Case. PLoS One. 2019;14(2):e0210497. doi: 10.1371/journal.pone.0210497 30807573 PMC6391029

[32] Choko AT, Candfield S, Maheswaran H, Lepine A, Corbett EL, Fielding K. The effect of demand-side financial incentives for increasing linkage into HIV treatment and voluntary medical male circumcision: A systematic review and meta-analysis of randomised controlled trials in low- and middle-income countries. PLoS One. 2018;13(11):e0207263. doi: 10.1371/journal.pone.0207263 30427889 PMC6235355

[33] Larson BA, Bii M, Halim N, Rohr JK, Sugut W, Sawe F. Incremental treatment costs for HIV-infected women initiating antiretroviral therapy during pregnancy: A 24-month micro-costing cohort study for a maternal and child health clinic in Kenya. PLoS One. 2018;13(8):e0200199. doi: 10.1371/journal.pone.0200199 30096177 PMC6086393

[34] UNAIDS. UNAIDS Data 2019 2019 [https://www.unaids.org/sites/default/files/media_asset/2019-UNAIDS-data_en.pdf.

