# Supplementary material for: The early-stage comprehensive costs of routine PrEP implementation and scale-up in Zambia
Source: PLOS Glob Public Health. 2022 Nov 2;2(11):e0001246. doi: 10.1371/journal.pgph.0001246 (PMC10021804; doi:10.1371/journal.pgph.0001246)
Supplement: S1 Table — (DOCX) [file pgph.0001246.s001.docx]

**S1 Table: 2021 costs of PrEP-provision according to Zambian guidelines (USD)**

| **Program Type** | **AGYW** | **FSW/MSM** | **Integrated 1** | **Integrated 2** | **Integrated 3** |
| --- | --- | --- | --- | --- | --- |
| **Number of PrEP clients initiated in program** | 908 | 213 | 322 | 214 | 148 |
| **Number of total sites** | 13 | 4 | 90 | 4 | 27 |
| **Average number of PrEP-clients/site** | 69.8 | 53.3 | 3.6 | 53.5 | 5.5 |
| **Start-up costs/site** |  |  |  |  |  |
| Community sensitization/consultation | 0 | 0 | 212 | 888 | 329 |
| Training | 5404 | 3861 | 907 | 418 | 218 |
| Site assessments | 0 | 0 | 0 | 573 | 0 |
| Equipment | 1560 | 1560 | 0 | 0 | 0 |
| **Average cost of start-up/site** | 6963 | 5421 | 1119 | 1879 | 547 |
| **Average cost of start-up/site/PrEP-client** | 100 | 102 | 313 | 35 | 100 |
| **Recurrent costs** |  |  |  |  |  |
| **Program support costs/PrEP-client** |  |  |  |  |  |
| Technical support | 0 | 0 | 0 | 210 | 360 |
| Training | 20 | 26 | 112 | 23 | 36 |
| PrEP-client support | 86 | 112 | 0 | 0 | 115 |
| PrEP provider support | 17 | 0 | 174 | 0 | 0 |
| Demand creation communication | 30 | 42 | 3 | 0 | 0 |
| **Average program support costs/PrEP-client (% of recurrent costs)** | 153 | 180 | 288 | 233 | 511 |
| **Direct service delivery costs/PrEP-client** |  |  |  |  |  |
| Staff & Overheads | 62 | 62 | 62 | 62 | 62 |
| Labs and monitoring | 87 | 93 | 84 | 84 | 84 |
| Consumables | 17 | 17 | 17 | 17 | 17 |
| PrEP Drugs | 117 | 117 | 117 | 117 | 117 |
| **Average direct service delivery costs/PrEP-client (% of recurrent costs)** | 282 | 288 | 279 | 279 | 279 |
| **Total recurrent cost/PrEP-client/year** | 435 | 469 | 567 | 512 | 790 |
| **Total average cost/PrEP-client/year** | 534 | 570 | 880 | 547 | 890 |
| **Total cost/person-month**** | 45 | 48 | 73 | 46 | 74 |

*Costs inflated from 2018 cost year using the cumulative CPI rate of 1.36

**Assuming 12 months effective PrEP coverage per PrEP-clientAbbreviations. PrEP: Pre-exposure Prophylaxis; USD: United States Dollar; AGYW: Adolescent girls and young women; MSM: Men-who-have-sex-with-men; FSW: Female Sex Worker.
